# Systemic Inflammation and Survival in Stage IV Colorectal Cancer: A Retrospective Cohort Study

**DOI:** 10.3390/jcm15062319

**Published:** 2026-03-18

**Authors:** Razvan Constantin Vonica, Nastaca Alina Palade, Anca Butuca, Vlad-Norin Vornicu, Claudiu Morgovan, Manuela Pumnea, Remus Calin Cipaian, Adina Frum, Florina Batar, Adelaida Solomon, Andreea Loredana Vonica-Tincu, Carmen Maximiliana Dobrea, Felicia Gabriela Gligor

**Affiliations:** 1 Preclinical Department, Faculty of Medicine, “Lucian Blaga” University of Sibiu, 550169 Sibiu, Romania; razvanconstantin.vonica@ulbsibiu.ro (R.C.V.); claudiu.morgovan@ulbsibiu.ro (C.M.); manuela-pia.pumnea@ulbsibiu.ro (M.P.); adina.frum@ulbsibiu.ro (A.F.); florina.batar@ulbsibiu.ro (F.B.); loredana.vonica@ulbsibiu.ro (A.L.V.-T.); carmen.dobrea@ulbsibiu.ro (C.M.D.); felicia.gligor@ulbsibiu.ro (F.G.G.); 2 Department IX Surgery, Discipline of Oncology, Faculty of Medicine, Victor Babes University of Medicine and Pharmacy Timisoara, Eftimie Murgu Square 2, 300041 Timisoara, Romania; vlad.vornicu@umft.ro; 3 Clinical Department, Faculty of Medicine, “Lucian Blaga” University of Sibiu, 550169 Sibiu, Romania; calin.cipaian@ulbsibiu.ro (R.C.C.); adelaida.nuta@ulbsibiu.ro (A.S.); 4 County Clinical Emergency Hospital of Sibiu, 2-4 Corneliu Coposu Str., 550245 Sibiu, Romania

**Keywords:** neutrophil-to-lymphocyte ratio (dNLR), colon cancer, personalized therapy, overall survival, systemic inflammation

## Abstract

**Background**: In metastatic colorectal cancer (mCRC), systemic inflammation and routine laboratory parameters may reflect host–tumor interactions and provide additional prognostic information. This study evaluated the association between baseline clinicopathological and laboratory parameters, including the derived neutrophil-to-lymphocyte ratio (dNLR), and overall survival (OS) in patients with stage IV colorectal adenocarcinoma. **Methods**: We conducted a retrospective cohort study including 92 patients diagnosed with metastatic colorectal adenocarcinoma and treated at a single oncology center between February 2022 and December 2024. Baseline laboratory parameters were collected at diagnosis. Survival was analyzed using Kaplan–Meier estimates with log-rank testing. Prognostic associations were evaluated using univariable and multivariable Cox proportional hazards regression models adjusted for relevant clinical and treatment-related factors. **Results**: The cohort was predominantly male (62%) and younger than 70 years (66%), with 80 deaths recorded during follow-up. In univariable analyses, primary tumor resection, irinotecan-based first-line therapy, elevated AST, and dNLR tertiles were associated with OS. However, after multivariable adjustment, only irinotecan-based first-line therapy remained independently associated with poorer survival (HR 2.10, 95% CI 1.16–3.81; *p* = 0.022). Continuous dNLR, anemia (WHO sex-specific), and AST elevation did not retain independent prognostic significance. **Conclusions**: In this cohort of patients with mCRC, inflammation-related laboratory markers demonstrated associations with survival in unadjusted analyses but did not remain independent predictors after adjustment for clinical and treatment-related confounders. These findings highlight the importance of rigorous multivariable modeling when evaluating inflammatory biomarkers in metastatic colorectal cancer.

## 1. Introduction

Colorectal cancer is a major cause of mortality and morbidity worldwide. In clinical oncology practice, risk assessment and survival assessment are quantified by tumor extension, tumor location, histological grade, and patient performance status. These criteria are the basis for personalized systemic treatment [1]. In the case of patients with metastatic disease, which is stage IV of the disease, tumor heterogeneity does not always conform to the traditional parameters, and the complexity of cases has interactions between the tumor and the host, with a significant impact on survival [2].

Neoplasia induces systemic inflammation, known as a key mechanism, with implications in tumor progression, angiogenesis, invasion and metastasis, with a particular response to treatment. In this regard, blood count indices and routine biochemical analysis can be considered as potential prognostic biomarkers, having a low cost, accessibility and reproducibility [3]. Among them, the neutrophil/lymphocyte index and its derived variants, including the neutrophil (leukocyte—neutrophil) ratio (dNLR), reflect the balance between immune-inflammatory activation and antitumor immune competence. Assessment of the inflammatory state at the time of diagnosis provides additional information on mortality risk and treatment response [4].

Also, the evaluation of treatment efficacy and safety is a central element of modern clinical practice, in which therapeutic decisions must be based not only on the demonstration of clinical benefit but also on a structured analysis of the risks associated with treatment. This approach is all the more relevant in the oncological context, where therapeutic decisions are directly related to survival, patient’s quality of life and management of adverse effects. In colorectal cancer, patient survival depends significantly on the stage of the disease at diagnosis, and for patients with stage IV disease, the goals of treatment include prolonging survival, maintaining quality of life, and reducing symptoms, in a context in which complete cure remains, in most cases, unattainable [5]. Standard treatment for metastatic disease includes combinations of chemotherapy based on 5-fluorouracil (5-FU), oxaliplatin (OX), and irinotecan (IRI), frequently associated with targeted therapies with monoclonal antibodies, such as bevacizumab (BEV) or panitumumab (PA) [6]. The results of these regimens are variable and depend on the biological characteristics of the tumor, the evolutionary stage of the disease, and the individual patient’s tolerability to treatment.

In addition to conventional therapeutic approaches, in recent years the emergence of integrated and personalized therapeutic strategies in the management of metastatic colorectal cancer has revolutionized oncology [7]. The median overall survival has improved significantly thanks to combined chemotherapy regimens and targeted therapies, including anti–vascular endothelial growth factor (anti-VEGF) and anti-epidermal growth factor receptor (anti-EGFR) monoclonal antibodies, which can be selected based on the molecular profile of the tumor [8]. For example, therapeutic response and survival can be influenced by specific genetic markers or molecular variants, which increasingly justify the use of biomarkers to personalize treatment and optimize clinical outcomes [9]. Such approaches not only increase efficacy but also reduce toxicities that are irrelevant to certain subgroups of patients, contributing to a more precise and focused management of the individual characteristics of the disease [10]. In addition, recent literature highlights the need for dynamic, multidimensional evaluations of treatment that incorporate both traditional clinical parameters and advanced biological markers and prediction algorithms. By correlating clinical data with these new tools, the quality of therapeutic decisions can be significantly improved and patients who would truly benefit from specific interventions can be identified, thus maximizing the benefit-to-risk ratio [11].

Careful monitoring of biological parameters during oncological treatment is fundamental for assessing both the efficacy and safety of therapy, enabling early identification of toxicities that may limit continuation of the therapeutic regimen. Hematological abnormalities (e.g., anemia and thrombocytopenia) or biochemical imbalances (such as increased total bilirubin, transaminases, and serum creatinine) may compromise patient tolerance and may lead to interruption or adjustment of therapy [12,13]. Such toxicities should be integrated into the benefit–risk assessment for each individual patient.

It is essential to highlight the importance of dynamic assessment of biological markers for optimizing personalized treatment in oncology. By precisely adjusting therapeutic regimens based on biomarker changes, therapeutic efficacy can be maintained while reducing the risk of severe adverse events. Correlating these parameters with overall survival and disease progression provides valuable prognostic information and supports the development of therapeutic strategies better tailored to the individual needs of patients with advanced colorectal cancer [14].

In this complex context, the present article aims to review the methodology and applicability of the main tools used in assessing the efficacy and safety of treatment, discussing how evidence from clinical trials and real-world data-based practices can be integrated to inform patient-centered and evidence-based therapeutic decisions. Based on these considerations, the present study also aims to explore the relationship between laboratory inflammatory markers determined at diagnosis and overall survival in patients with stage IV colorectal adenocarcinoma, in active oncological treatment. In a retrospective cohort setting, clinicopathological data and baseline hematological and biochemical parameters, with a focus on dNLR, were analyzed in order to identify associations with disease characteristics and their prognostic potential on survival.

## 2. Materials and Methods

This study was approved by the Ethics Committee of the OncoHelp Oncology Center, Timișoara, Romania (No. 434/7 March 2025), and by the Ethics Committee of Lucian Blaga University of Sibiu, Romania (No. 3960/11 July 2025).

### 2.1. Study Design and Patient Selection

**Figure 1:**
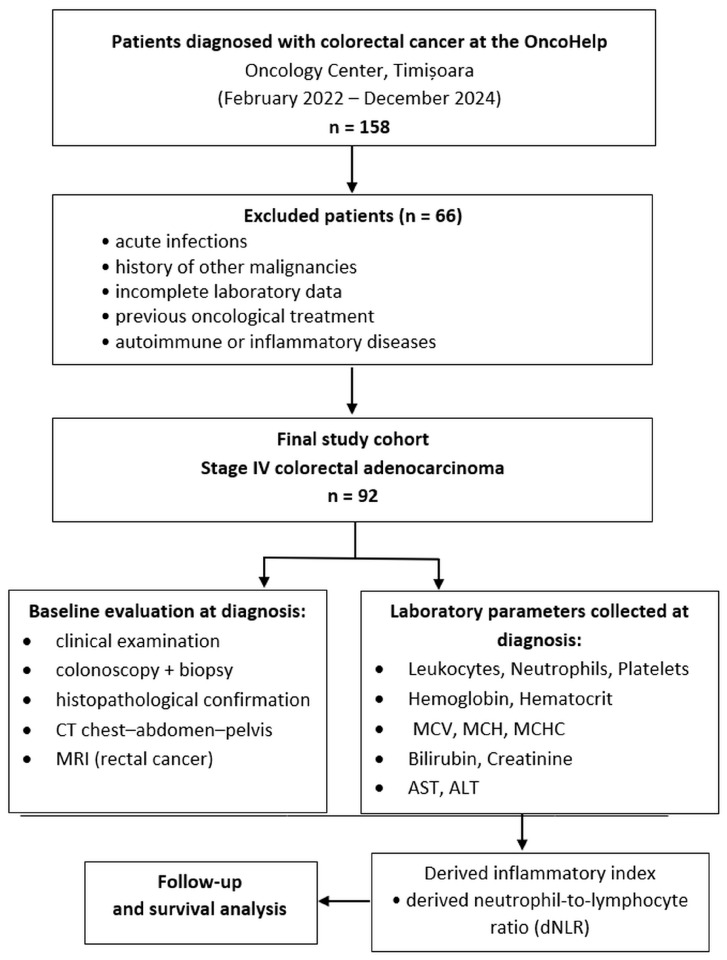
Colorectal cancer patients’ selection process.

This retrospective cohort study included patients with stage IV colorectal adenocarcinoma diagnosed at the OncoHelp Oncology Center, Timisoara, Romania, between February 2022 and December 2024. Initially, 158 patients were screened for eligibility. After the application of predefined inclusion and exclusion criteria, 92 patients were included in the final analysis (Figure 1). Patients were enrolled at the time of their first presentation to the oncology outpatient clinic.

At diagnosis, all patients underwent a comprehensive clinical evaluation, including physical examination, routine laboratory testing, colonoscopy with biopsy followed by histopathological confirmation, and radiological assessment for disease staging. Imaging evaluation included contrast-enhanced computed tomography (CT) of the chest, abdomen, and pelvis. Magnetic resonance imaging (MRI) was additionally performed in patients with rectal cancer when clinically indicated.

The inclusion criteria were as follows: (i) newly diagnosed colorectal adenocarcinoma, (ii) stage IV disease at diagnosis, (iii) availability of complete baseline laboratory data obtained at diagnosis, and (iv) absence of prior oncological treatment.

The exclusion criteria included: acute infections at diagnosis, autoimmune or inflammatory diseases potentially affecting laboratory parameters, chronic systemic corticosteroid therapy, prior chemotherapy or biological treatment, and a personal history of other malignancies.

Baseline laboratory parameters were obtained at the time of diagnosis using standard automated hematology and biochemistry analyzers. Reference ranges for all laboratory variables were defined according to institutional laboratory standards and are reported in the Supplementary Materials (Table S1). The laboratory parameters analyzed included hematocrit (HCT), hemoglobin (HGB), mean corpuscular volume (MCV), mean corpuscular hemoglobin (MCH), mean corpuscular hemoglobin concentration (MCHC), platelet count (PLT), white blood cell count (WBC), absolute neutrophil count (ANC), aspartate aminotransferase (AST), alanine aminotransferase (ALT), total bilirubin, and serum creatinine.

The derived neutrophil-to-lymphocyte ratio (dNLR) was calculated as ANC/(WBC − ANC) and subsequently analyzed as an inflammation-based prognostic marker.

### 2.2. Performance Status Assessment (ECOG)

Performance status was assessed at baseline using the Eastern Cooperative Oncology Group (ECOG) performance status scale, a widely used clinical tool for evaluating patients’ functional status and ability to perform daily activities. The ECOG scale ranges from 0 to 5, where 0 indicates fully active patients without functional limitations, and higher scores reflect increasing levels of disability, with 5 corresponding to death.

ECOG performance status was determined by the treating physician at the time of diagnosis, based on clinical evaluation and patient-reported functional capacity. For analytical purposes, ECOG performance status was evaluated as a categorical variable.

### 2.3. Statistical Analysis

Statistical analysis was performed to evaluate the association between baseline laboratory inflammatory markers and clinicopathological characteristics, as well as their prognostic impact on overall survival in patients with colorectal cancer. The analyzed clinicopathological variables included age, sex, primary tumor location, TNM stage (T, N, and M1), pathological tumor grade, performance status, and survival status.

Data management, statistical analyses, and graphical representations were conducted using Microsoft Excel 2021 (Microsoft Corp., Redmond, WA, USA) and IBM SPSS Statistics version 26 (IBM Corp., Armonk, NY, USA). Continuous variables were tested for normality using the Shapiro–Wilk test. Normally distributed data were expressed as mean ± standard deviation, while non-normally distributed data were reported as median and interquartile range (IQR).

Comparisons between two independent groups were performed using the independent samples Student’s *t*-test for normally distributed variables or the Mann–Whitney U test for non-normally distributed variables. For comparisons among more than two groups, one-way analysis of variance (ANOVA) followed by Tukey’s post hoc test was applied for normally distributed data, while the Kruskal–Wallis test followed by Dunn’s post hoc test was used for non-normally distributed data.

Overall survival (OS) was defined as the time from diagnosis to death from any cause or last follow-up. Survival curves were estimated using the Kaplan–Meier method and compared using the log-rank test. Univariable Cox proportional hazards regression was performed to assess associations with OS. Variables of clinical relevance were subsequently entered into a multivariable Cox model adjusted for age, sex, ECOG performance status, primary tumor sidedness, liver metastases, primary tumor resection, first-line treatment class, anemia, AST, and dNLR. Hazard ratios (HRs) with 95% confidence intervals (CIs) were reported, and a two-sided *p*-value < 0.05 was considered statistically significant.

All statistical tests were two-sided, and a *p*-value < 0.05 was considered statistically significant.

## 3. Results

The study cohort comprised eligible patients with stage IV colorectal adenocarcinoma. Most patients were younger than 70 years (66.37%), while 33.63% were aged 70 years or older. The study population was predominantly male, with men accounting for 62% of cases and women for 38%. Regarding tumor location, left-sided colorectal tumors were more frequent, representing 76.1% of cases, whereas right-sided tumors accounted for 23.9%. Tumor staging was performed according to the Tumor–Node–Metastasis (TNM) classification system, which evaluates the extent of the primary tumor (T), regional lymph node involvement (N), and the presence of distant metastases (M1). According to tumor extent, patients were almost equally distributed between T3 (59.8%) and T4 (40.2%) stages (Table 1).

Lymph node involvement was common, with the majority of patients presenting nodal metastases (N1–N2, 80.4%), while 19.6% had no lymph node involvement (N0). As expected for a stage IV cohort, distant metastases were present in all included patients (M1, 100%). Regarding pathological differentiation, moderately differentiated tumors (G2) were the most prevalent (52.20%), followed by poorly differentiated tumors (G3, 22.83%) and well-differentiated tumors (G1, 25.07%). Performance status assessment showed that most patients had a good functional status, with ECOG (Tables S2 and S3) performance index scores of 0 or 1 in 94.6% of cases, while only 5.4% had a performance status of 2.

The median follow-up, calculated using the reverse Kaplan–Meier method, was 687 days (95% CI: 647–727). No patients were lost to follow-up. Survival status was ascertained through the institutional oncology registry. Initial systemic treatment consisted mainly of combination chemotherapy regimens associated with targeted agents. First-line systemic treatment was most commonly oxaliplatin-based (46.7%), followed by irinotecan-based regimens (29.3%) and fluoropyrimidine-based therapies (23.9%).

### 3.1. Association of Baseline Laboratory Parameters and Derived Inflammatory Indices with Clinicopathological Characteristics and Outcomes

#### 3.1.1. Association of Inflammatory Markers with TNM Stage

When laboratory parameters were compared according to primary tumor extent, no statistically significant differences were observed between T3 and T4 tumors for most hematological and biochemical variables (Table 2). Hemoglobin-related parameters, leukocyte counts, liver enzymes, renal function markers, and the derived inflammatory index (dNLR) showed comparable values between the two groups. These findings suggest that, within an advanced disease setting, local tumor extension alone does not substantially influence systemic inflammatory or biochemical parameters measured at diagnosis.

Across lymph node categories, hematological and biochemical parameters remained largely stable (Table 3). No significant differences were observed in hemoglobin levels, erythrocyte indices, renal or hepatic markers. The dNLR ratio also showed no significant variation across N stages. Platelet count differed significantly across lymph node stages (overall *p*-value = 0.011), with higher values in N1 and N2 compared to N0. Dunn’s post hoc analysis showed significant differences for N0 vs. N1 (*p*-value = 0.045) and N0 vs. N2 (*p*-value = 0.043), but not for N1 vs. N2 (*p*-value = 0.125). This finding suggests a potential association between thrombocytosis and lymphatic tumor spread. White blood cell and neutrophil counts remained relatively stable across N stages, indicating that nodal disease burden alone may not substantially influence systemic inflammatory activation at diagnosis.

The hematological and biochemical characteristics of the M1 cohort (n = 92) are presented in Table 4.

#### 3.1.2. Association of Inflammatory Markers with Tumor Pathological Grade

Baseline laboratory parameters were compared according to histological grade (G1–G3). No statistically significant differences were observed in most hematological and biochemical markers across tumor grades. Hemoglobin, hematocrit, erythrocyte indices (MCH, MCHC, MCV), platelet count, total bilirubin, serum creatinine, ANC, AST, ALT, and dNLR showed comparable distributions between G1, G2, and G3 tumors (all *p* > 0.05). Although a trend toward higher leukocyte counts was observed in patients with G3 tumors, this did not reach statistical significance (*p* = 0.056). Similarly, dNLR values were not significantly different across histological grades (*p* = 0.860), suggesting that systemic inflammatory status at baseline was not directly associated with tumor differentiation grade in this cohort.

Overall, these findings indicate that baseline inflammatory and routine laboratory parameters were largely independent of histological grading in metastatic colorectal cancer (Table 5).

#### 3.1.3. Association of Laboratory Parameters with Performance Status

Performance status was significantly associated with hematological and inflammatory parameters (Table 6). Hematocrit levels showed a decreasing trend across performance index categories, although this did not reach statistical significance (*p* = 0.128). Total leukocyte count, absolute neutrophil count, and dNLR differed significantly according to performance index (WBC: *p* = 0.008; ANC: *p* = 0.003; dNLR: *p* = 0.001). Post hoc pairwise comparisons using Dunn’s test demonstrated significantly higher values in P1 and P2 compared with P0 (WBC: P1 vs. P0, adjusted *p*-value = 0.018; P2 vs. P0, adjusted *p*-value = 0.023; ANC: P1 vs. P0, adjusted *p*-value = 0.010; P2 vs. P0, adjusted *p*-value = 0.036; dNLR: P1 vs. P0, adjusted *p*-value = 0.010; P2 vs. P0, adjusted *p*-value = 0.011), whereas no significant differences were observed between P1 and P2 (all adjusted *p*-value > 0.10). ALT levels showed a non-significant trend across performance categories (*p* = 0.064). Other hematological and biochemical variables did not show statistically significant differences.

#### 3.1.4. Association of Inflammatory Markers with Demographic Characteristics and Primary Tumor Location

Clinicopathological characteristics, including age, sex, and primary tumor location were evaluated in relation to baseline inflammatory and laboratory parameters (Table 7).

When stratified by age, several laboratory parameters differed between patients younger than 70 years and those aged 70 years or older. Serum creatinine levels were significantly higher in older patients (*p*-value = 0.031), while the derived neutrophil-to-lymphocyte ratio was also increased in this age group (*p*-value = 0.035). Other hematological parameters, including hemoglobin, leukocyte counts, absolute neutrophil counts, platelet counts, and liver enzymes, showed comparable values between age categories. These findings suggest that age-related differences were mainly reflected in renal function and inflammatory balance rather than in global hematological profiles.

According to sex, significant differences were observed in several inflammation related parameters. White blood cell counts were significantly higher in men compared to women (*p*-value = 0.038), accompanied by increased absolute neutrophil counts (*p*-value = 0.034). In addition, serum creatinine levels were significantly higher in male patients (*p*-value < 0.001). Total bilirubin levels were higher in women (*p*-value = 0.046), while the derived neutrophil-to-lymphocyte ratio did not differ significantly between sexes. Other haematological and biochemical parameters showed no statistically significant differences.

When patients were stratified according to primary tumor location, most laboratory parameters showed similar distributions between left- and right-sided colorectal tumors. Mean corpuscular volume was significantly higher in patients with left-sided tumors (*p*-value = 0.048). Absolute neutrophil counts and derived neutrophil-to-lymphocyte ratio tended to be higher in right-sided tumors; however, these differences did not reach statis-tical significance. No significant differences were observed for platelet counts, leukocyte counts, liver enzymes, renal function markers, or total bilirubin according to tumor loca-tion.

Overall, demographic factors such as age and sex were associated with selective al-terations in inflammatory and biochemical parameters, while primary tumor location showed a limited influence on baseline systemic inflammatory profiles in this cohort.

### 3.2. Kaplan–Meier Analysis of Overall Survival According to Inflammatory Marker Levels

**Figure 2:**
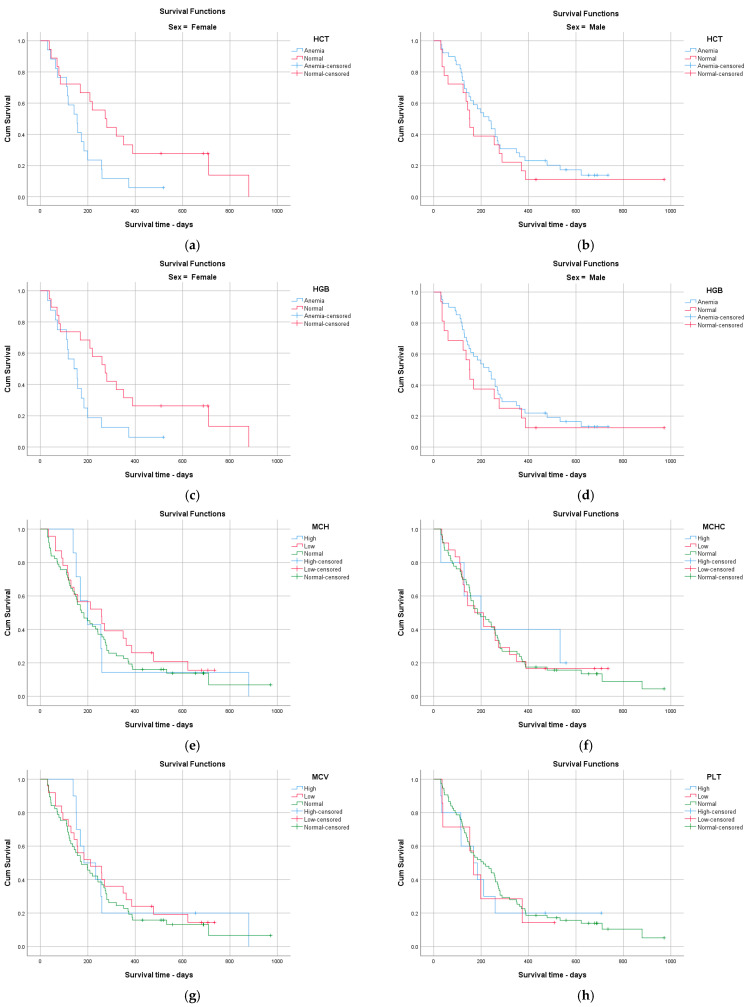
Kaplan–Meier overall survival curves according to baseline hematological and biochemical parameters: HCT—female (**a**), HCT—male (**b**), HGB—female (**c**), HGB—male (**d**) MCH (**e**), MCHC (**f**), MCV (**g**), PLT (**h**).

**Figure 3:**
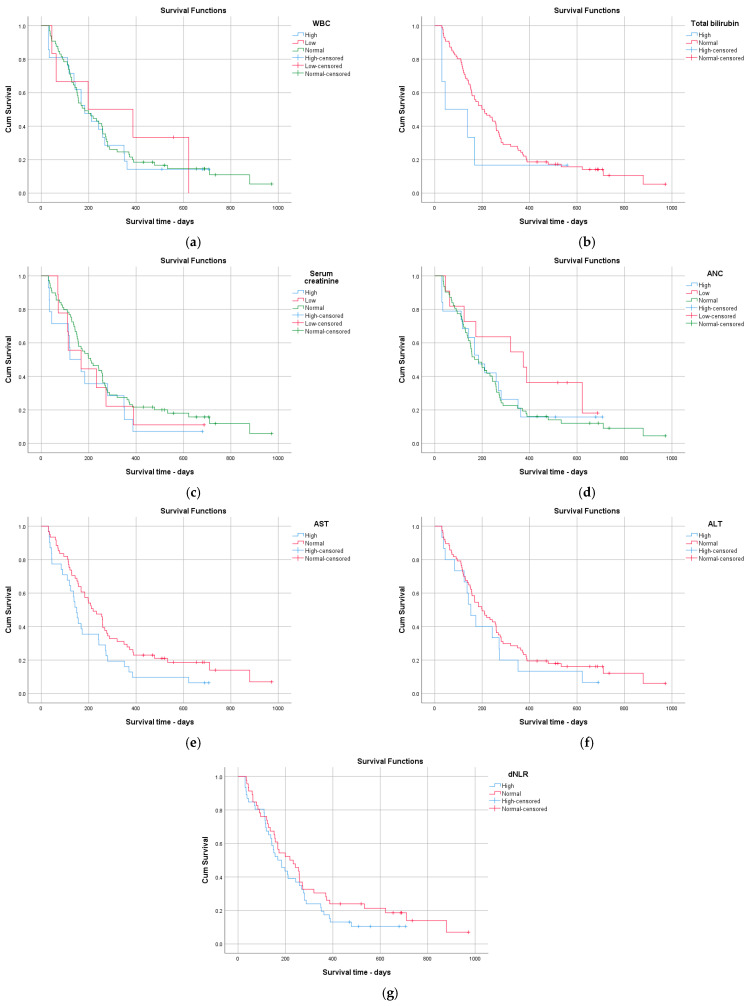
Kaplan–Meier survival analyses were performed for baseline inflammatory and biochemical markers, including WBC (**a**), total bilirubin (**b**), serum creatinine (**c**), ANC (**d**), AST (**e**), ALT (**f**), and dNLR (**g**).

In Kaplan–Meier analysis, only AST elevation was significantly associated with shorter overall survival. Other routine hematological and biochemical parameters, including hemoglobin, hematocrit, platelet count, leukocyte count, bilirubin, creatinine, and dNLR (median-based cut-off), were not significantly associated with survival. Although some markers showed numerical differences in median OS, these did not reach statistical significance, likely reflecting limited subgroup sizes and clinical heterogeneity (Figure 2 and Figure 3, Table 8).

### 3.3. Cox Regression Mode

In univariable Cox regression analysis, primary tumor resection and irinotecan-based first-line therapy were significantly associated with overall survival. Patients who underwent primary tumor resection had improved survival (HR 0.609, 95% CI 0.373–0.995, *p* = 0.048). In contrast, irinotecan-based regimens were associated with increased mortality risk compared with fluoropyrimidine-only therapy (HR 1.810, 95% CI 1.036–3.165, *p* = 0.037). Elevated AST showed borderline statistical significance (HR 1.588, 95% CI 1.001–2.519, *p* = 0.050). Continuous dNLR was not significantly associated with survival; however, patients in the second tertile (T2) had significantly worse survival compared with T1 (HR 1.983, 95% CI 1.152–3.413, *p* = 0.014). No significant associations were observed for age, sex, ECOG performance status, tumor sidedness, liver metastases, anemia, or oxaliplatin-based therapy.

After adjustment for clinically relevant confounders, only irinotecan-based first-line therapy remained independently associated with overall survival (HR 2.103, 95% CI 1.162–3.808, *p* = 0.022). The associations observed in univariable analysis for primary tumor resection, AST, and dNLR did not persist in the multivariable model. Specifically, AST elevation (HR 1.234, 95% CI 0.947–1.608, *p* = 0.119), anemia (HR 1.420, 95% CI 0.865–2.306, *p* = 0.165), and continuous dNLR (HR 1.117, 95% CI 0.849–1.470, *p* = 0.429) were not independently associated with survival.

In this cohort of metastatic colorectal cancer patients, irinotecan-based therapy was the only independent predictor of overall survival. The prognostic associations observed for AST and dNLR in unadjusted analyses were attenuated after adjustment for clinicopathological and treatment-related factors (Table 9).

## 4. Discussion

In this retrospective cohort of patients with metastatic colorectal adenocarcinoma, we investigated the prognostic impact of baseline clinicopathological characteristics and routine laboratory parameters on overall survival. Although several variables were associated with survival in univariable analyses, only irinotecan-based first-line therapy remained independently associated with overall survival after adjustment for major clinical confounders.

Systemic inflammation has been extensively studied in metastatic colorectal cancer. In our cohort, dNLR was associated with performance status and certain pathological features, and patients in the intermediate tertile demonstrated worse survival in univariable analysis. However, continuous dNLR did not retain independent significance in the multivariable model. These findings suggest that systemic inflammatory indices may partially reflect disease burden and host condition rather than acting as fully independent prognostic determinants. Large prospective trials, including COIN and TRIBE, have reported independent associations between elevated NLR/dNLR and inferior survival in mCRC [15,16,17]. Our results support the biological relevance of inflammation but underscore the importance of comprehensive adjustment for metastatic pattern, functional status, and treatment allocation when evaluating inflammatory biomarkers [18,19,20].

Similarly, elevated AST was associated with shorter survival in unadjusted analyses and Kaplan–Meier comparisons. However, this effect was attenuated after multivariable adjustment. In metastatic colorectal cancer, AST elevation may reflect hepatic metastatic involvement, systemic inflammatory activation, metabolic stress, or muscle catabolism rather than isolated hepatocellular injury [21,22,23]. The loss of independent significance indicates that AST likely functions as a surrogate marker of disease extent rather than an autonomous prognostic factor.

Anemia, defined according to sex-specific WHO criteria, was also associated with survival in univariable analysis but did not remain significant in the adjusted model. Cancer-related anemia is multifactorial and frequently driven by chronic inflammation, iron dysregulation, nutritional impairment, and bone marrow suppression [24,25,26]. Its attenuation after adjustment suggests that hemoglobin levels may represent overall disease severity and physiological reserve rather than exerting an independent prognostic effect.

Notably, irinotecan-based first-line therapy remained independently associated with shorter survival. This finding should be interpreted cautiously, as treatment selection in metastatic colorectal cancer is not randomized and may reflect confounding by indication. Patients receiving irinotecan-based regimens may have had more aggressive disease biology or clinical features influencing therapeutic decisions. Similar considerations have been described in observational studies evaluating treatment patterns in mCRC [27,28]. Therefore, the observed association likely reflects underlying disease complexity rather than a direct detrimental effect of irinotecan itself.

Across histological grade, T stage, nodal status, and performance categories, laboratory parameters showed heterogeneous but generally modest differences. Although inflammatory markers tended to increase with worsening performance status, these variations did not translate into independent survival effects once clinical and treatment-related confounders were considered. Other routine laboratory parameters—including platelet count, leukocyte count, bilirubin, creatinine, and ALT—were not independently associated with survival, in line with prior evidence suggesting that composite inflammatory indices may outperform isolated biomarkers [28,29,30].

Overall, our findings highlight the complexity of prognostic modeling in metastatic colorectal cancer. While inflammation-based markers such as dNLR and AST demonstrate associations with survival in unadjusted analyses, their independent prognostic contribution appears limited after accounting for key clinical and treatment variables. These results emphasize the necessity of rigorous multivariable modeling in biomarker evaluation.

Several limitations merit consideration. The retrospective character implies the use of existing data in observation sheets, which can lead to incomplete information, variability in the documentation of clinical parameters and potential recording errors. The single-center design and relatively small sample size may limit statistical power and increase susceptibility to residual confounding. Moreover, only baseline laboratory parameters were analyzed; dynamic changes during treatment could provide additional prognostic insight [31]. Consequently, the results should be interpreted with caution and validated by multicenter, prospective studies in larger batches of patients. Nevertheless, the internal consistency of our findings and their alignment with contemporary literature support the role of systemic inflammatory and metabolic markers as reflections of disease burden in metastatic colorectal cancer.

## 5. Conclusions

In this cohort of patients with metastatic colorectal adenocarcinoma, several inflammation-related and routine laboratory parameters were associated with overall survival in unadjusted analyses. However, after adjustment for clinicopathological and treatment-related confounders, only irinotecan-based first-line therapy remained independently associated with survival. Although markers such as dNLR and AST demonstrated prognostic signals in univariable analyses, their effects were attenuated in multivariable modeling, suggesting that these biomarkers may primarily reflect disease burden and host condition rather than acting as autonomous prognostic determinants.

These findings underscore the importance of comprehensive multivariable adjustment when evaluating inflammation-based biomarkers in metastatic colorectal cancer. Further prospective studies with larger cohorts and dynamic longitudinal assessment of inflammatory indices are warranted to clarify their independent prognostic value.

## Figures and Tables

**Table 1:** Baseline characteristics and initial treatment of patients with colorectal adenocarcinoma.

Patient Characteristics		No. of Cases
Age	≥70 years old	31 (33.63%)
	<70 years old	61 (66.37%)
Gender	Male	57 (62%)
	Female	35 (38%)
Tumor Localization	Left Colon	70 (76.1%)
	Right Colon	22 (23.9%)
T Stage	T3	55 (59.8%)
	T4	37 (40.2%)
N Stage	N0	18 (19.6%)
	N1	38 (41.3%)
	N2	36 (39.1%)
M Stage	M1	92 (100%)
G Grade	G1	23 (25.0%)
	G2	48 (52.2%)
	G3	21 (22.8%)
P Intex	P0	30 (32.6%)
	P1	57 (62.0%)
	P2	5 (5.4%)
Liver metastases	Yes	75 (81.5)
	No	17 (18.5)
Primary resection	Yes	67 (72.8)
	No	25 (27.2)
Initial treatment	Fluoropyrimidine-base	22 (23.9%)
	Oxaliplatin-based	43 (46.7%)
	Irinotecan-based	27 (29.3%)

Note: T indicates the extent of the primary tumor (T3, tumor invasion into pericolonic/perirectal tissues; T4, tumor invasion of adjacent organs or visceral peritoneum); N indicates regional lymph node involvement (N0, no nodal metastases; N1, 1–3 positive nodes; N2, ≥4 positive nodes); M1 indicates the presence of distant metastases. G = histological tumor grade (G1, well differentiated; G2, moderately differentiated; G3, poorly differentiated). P (Performance Index) = patient functional status assessed at baseline (P0, fully active; P1, restricted in physically strenuous activity; P2, ambulatory and capable of self-care but unable to carry out work activities).

**Table 2:** Comparison of baseline laboratory parameters according to primary tumor extent (T stage).

Tumor Invasion	T3	T4	*p*-Value
No. of Patients	55	37	
Marker			
HCT	36.57 ± 4.98	35.51 ± 4.62	0.317 ^(a)^
HGB	12.01 ± 1.93	11.75 ± 1.57	0.495 ^(a)^
MCH	27.49 ± 3.59	28.31 ± 3.45	0.276 ^(a)^
MCHC	32.90 (32.25, 33.35)	33.10 (32.20, 33.60)	0.311 ^(b)^
MCV	83.59 ± 8.99	85.39 ± 9.11	0.351 ^(a)^
PLT	280.00 (232.00, 363.50)	281.00 (212.00, 371.00)	0.577 ^(b)^
WBC	7.46 (6.16, 9.45)	7.82 (5.60, 10.51)	0.949 ^(b)^
total bilirubin	0.57 (0.41, 0.74)	0.59 (0.45, 0.69)	0.845 ^(b)^
serum creatinine	0.79 ± 0.24	0.71 ± 0.17	0.061 ^(a)^
ANC	4.64 (2.88, 6.94)	4.90 (2.64, 6.57)	0.962 ^(b)^
AST	28.94 (22.95, 42.63)	29.79 (19.17, 43.33)	0.705 ^(b)^
ALT	20.36 (13.32, 29.22)	19.15 (13.20, 30.30)	0.911 ^(b)^
dNLR	1.57 (1.16, 2.60)	1.64 (1.03, 2.14)	0.442 ^(b)^

Note: Data are presented as mean ± standard deviation or median (interquartile range), as appropriate. Comparisons between groups were performed using the independent samples *t*-test ^(a)^ or the Mann–Whitney U test ^(b)^; T3 (tumor invasion into pericolonic/perirectal tissues); T4 (tumor invasion of adjacent organs or visceral peritoneum); HCT (hematocrit); HGB (hemoglobin); MCV (mean corpuscular volume); MCH (mean corpuscular hemoglobin); MCHC (mean corpuscular hemoglobin concentration); PLT (platelet count); WBC (white blood cell count); ANC (absolute neutrophil count); AST (aspartate aminotransferase); ALT (alanine aminotransferase); dNLR (calculated as ANC/[WBC − ANC]);

**Table 3:** Baseline laboratory parameters according to lymph node involvement (N stage).

Tumor Invasion	N0	N1	N2	*p*-Value
No. of Patients	18	38	36	
Marker				
HCT	36.52 ± 5.16	37.33 ± 5.19	34.71 ± 4.35	0.070 ^(a)^
HGB	12.04 ± 1.78	12.30 ± 1.89	11.43 ± 1.62	0.101 ^(a)^
MCH	28.63 ± 3.93	27.85 ± 3.33	27.38 ± 3.46	0.476 ^(a)^
MCHC	33.25 (32.70, 33.50)	33.00 (32.30, 33.50)	32.95 (32.15, 33.55)	0.829 ^(b)^
MCV	86.66 ± 10.13	84.49 ± 8.68	82.96 ± 8.82	0.365 ^(a)^
PLT	239.50 (211.00, 380.00)	298.00 (239.00, 361.00)	289.00 (214.50, 383.50)	**0.011** ^(b)^
WBC	7.48 (6.09, 9.23)	7.76 (5.31, 8.96)	7.64 (6.24, 10.84)	0.901 ^(b)^
total bilirubin	0.62 (0.47, 0.89)	0.53 (0.40, 0.70)	0.57 (0.44, 0.70)	0.182 ^(b)^
serum creatinine	0.79 ± 0.23	0.74 ± 0.19	0.77 ± 0.23	0.687 ^(a)^
ANC	4.77 (3.17, 7.12)	4.58 (2.64, 6.72)	4.80 (2.81, 6.72)	0.899 ^(b)^
AST	29.44 (25.22, 37.32)	29.21 (22.56, 43.84)	28.33 (19.78, 37.74)	0.970 ^(b)^
ALT	16.53 (12.97, 27.90)	20.70 (13.20, 30.76)	20.73 (13.28, 29.28)	0.702 ^(b)^
dNLR	1.62 (1.10, 2.37)	1.61 (1.12, 2.14)	1.81 (1.16, 2.58)	0.612 ^(b)^

Note: Data are presented as mean ± standard deviation or median (interquartile range), as appropriate. Comparisons between groups were performed using one-way ANOVA ^(a)^ or the Kruskal–Wallis test ^(b)^. Bold values indicate statistical significance (*p* < 0.05). N0 (no nodal metastases); N1 (1–3 positive nodes); N2 (≥4 positive nodes); HCT (hematocrit); HGB (hemoglobin); MCV (mean corpuscular volume); MCH (mean corpuscular hemoglobin); MCHC (mean corpuscular hemoglobin concentration); PLT (platelet count); WBC (white blood cell count); ANC (absolute neutrophil count); AST (aspartate aminotransferase); ALT (alanine aminotransferase); dNLR (calculated as ANC/[WBC − ANC]);

**Table 4:** Baseline Hematological and Biochemical Parameters in Patients with Stage IV Colorectal Cancer (M1, n = 92).

Tumor Invasion	M1
No. of Patients	92
Marker	
HCT	36.15 ± 4.96
HGB	11.91 ± 1.79
MCH	27.82 ± 3.54
MCHC	33.00 (32.20, 33.50)
MCV	84.31 ± 9.03
PLT	280.50 (220.50, 372.00)
WBC	7.70 (6.06, 9.55)
total bilirubin	0.57 (0.43, 0.73)
serum creatinine	0.76 ± 0.221
ANC	4.70 (2.86, 6.79)
AST	28.74 (21.90, 40.55)
ALT	19.76 (13.09, 29.67)
dNLR	1.63 (1.13, 2.43)

Note: Data are presented as mean ± standard deviation or median (interquartile range), as appropriate; or median (interquartile range), as appropriate. M1 indicates the presence of distant metastases; HCT (hematocrit); HGB (hemoglobin); MCV (mean corpuscular volume); MCH (mean corpuscular hemoglobin); MCHC (mean corpuscular hemoglobin concentration); PLT (platelet count); WBC (white blood cell count); ANC (absolute neutrophil count); AST (aspartate aminotransferase); ALT (alanine aminotransferase); dNLR (calculated as ANC/[WBC − ANC]).

**Table 5:** Baseline laboratory parameters according to tumor pathological grade (G stage).

Tumor Invasion	G1	G2	G3	*p*-Value
No. of Patients	23	48	21	
Marker				
HCT	36.10 (33.70, 38.65)	36.65 (32.85, 40.35)	32.80 (31.30, 39.10)	0.213 ^(b)^
HGB	11.50 (11.15, 12.55)	12.20 (10.55, 13.40)	10.60 (9.90, 12.60)	0.294 ^(b)^
MCH	27.11 ± 3.51	28.37 ± 3.35	27.33 ± 3.92	0.293 ^(a)^
MCHC	32.88 ± 1.25	32.96 ± 1.31	32.95 ± 1.34	0.967 ^(a)^
MCV	82.42 ± 8.54	85.92 ± 8.78	82.73 ± 9.85	0.207 ^(a)^
PLT	279.00 (244.00, 362.50)	271.00 (215.50, 368.00)	312.00 (213.00, 374.00)	0.707 ^(b)^
WBC	7.27 (6.31, 8.44)	7.17 (4.74, 9.41)	8.23 (7.70, 11.70)	0.056 ^(b)^
total bilirubin	0.56 (0.43, 0.70)	0.57 (0.45, 0.75)	0.61 (0.39, 0.71)	0.791 ^(b)^
serum creatinine	0.81 (0.68, 0.86)	0.69 (0.60, 0.86)	0.79 (0.65, 0.96)	0.260 ^(b)^
ANC	4.64 (3.42, 5.42)	4.18 (2.42, 6.80)	5.53 (4.52, 7.00)	0.144 ^(b)^
AST	31.55 (24.35, 39.18)	27.56 (21.59, 43.59)	28.50 (14.86, 36.76)	0.244 ^(b)^
ALT	16.66 (11.57, 28.31)	20.81 (15.351, 28.42)	20.36 (11.68, 30.30)	0.695 ^(b)^
dNLR	1.57 (1.21, 2.23)	1.67 (1.06, 2.56)	1.65 (1.21, 2.37)	0.860 ^(b)^

Note: Data are presented as mean ± standard deviation or median (interquartile range), as appropriate. Comparisons between groups were performed using one-way ANOVA ^(a)^ or the Kruskal–Wallis test ^(b)^; G is histological tumor grade (G1, well differentiated; G2, moderately differentiated; G3, poorly differentiated); HCT (hematocrit); HGB (hemoglobin); MCV (mean corpuscular volume); MCH (mean corpuscular hemoglobin); MCHC (mean corpuscular hemoglobin concentration); PLT (platelet count); WBC (white blood cell count); ANC (absolute neutrophil count); AST (aspartate aminotransferase); ALT (alanine aminotransferase); dNLR (calculated as ANC/[WBC − ANC]).

**Table 6:** Baseline laboratory parameters according to performance status (P stage).

Tumor Invasion	P0	P1	P2	*p*-Value
No. of Patients	30	57	5	
Marker				
HCT	37.65 ± 5.30	35.46 ± 4.62	35.00 ± 5.71	0.128 ^(a)^
HGB	12.31 ± 1.95	11.74 ± 1.69	11.42 ± 1.78	0.305 ^(a)^
MCH	28.08 ± 3.43	27.85 ± 3.71	25.86 ± 1.07	0.430 ^(a)^
MCHC	33.00 (32.20, 33.20)	33.10 (32.30, 33.60)	32.30 (32.20, 32.70)	0.354 ^(b)^
MCV	85.74 ± 9.30	84.00 ± 9.13	79.24 ± 3.38	0.305 ^(a)^
PLT	281.50 (217.00, 330.00)	280.00 (221.00, 373.00)	281.00 (277.00, 501.00)	0.665 ^(b)^
WBC	6.24 (4.61, 8.16)	7.92 (6.51, 10.28)	8.41 (8.00, 12.59)	**0.008** ^(b)^
total bilirubin	0.57 (0.37, 0.70)	0.58 (0.47, 0.74)	0.47 (0.46, 0.59)	0.497 ^(b)^
serum creatinine	0.71 (0.64, 0.86)	0.75 (0.60, 0.86)	0.78 (0.66, 0.96)	0.897 ^(b)^
ANC	2.70 (2.15, 5.33)	5.15 (3.55, 7.00)	6.29 (4.90, 10.55)	**0.003** ^(b)^
AST	29.32 (22.77, 43.95)	28.50 (22.17, 38.45)	32.10 (21.55, 35.86)	0.943 ^(b)^
ALT	23.90 (15.38, 42.81)	16.40 (12.47, 26.07)	25.71 (15.92, 26.26)	0.064 ^(b)^
dNLR	1.17 (0.70, 2.07)	1.82 (1.24, 2.57)	2.97 (1.95, 3.97)	**0.001** ^(b)^

Note: Data are presented as mean ± standard deviation or median (interquartile range), as appropriate. Comparisons between groups were performed using one-way ANOVA ^(a)^ or the Kruskal–Wallis test ^(b)^; Bold values indicate statistical significance (*p* < 0.05); P (Performance Index) = patient functional status assessed at baseline (P0, fully active; P1, restricted in physically strenuous activity; P2, ambulatory and capable of self-care but unable to carry out work activities); HCT (hematocrit); HGB (hemoglobin); MCV (mean corpuscular volume); MCH (mean corpuscular hemoglobin); MCHC (mean corpuscular hemoglobin concentration); PLT (platelet count); WBC (white blood cell count); ANC (absolute neutrophil count); AST (aspartate aminotransferase); ALT (alanine aminotransferase); dNLR (calculated as ANC/[WBC − ANC]).

**Table 7:** Baseline inflammatory marker levels stratified by age, sex, and primary tumor location.

	Age			Gender			Primary Tumor Location		
Marker	<70	≥70	*p*-Value	Woman	Men	*p*-Value	Left	Right	*p*-Value
HCT	36.20 ± 4.95	35.61 ± 4.65	0.565	36.03 ± 4.80	35.99 ± 4.90	0.964	36.23 ± 4.91	35.34 ± 4.66	0.427
HGB	11.91 ± 1.78	11.79 ± 1.66	0.751	11.86 ± 1.67	11.88 ± 1.78	0.965	11.96 ± 1.76	11.61 ± 1.64	0.381
MCH	27.65 ± 3.64	28.23 ± 3.02	0.427	28.09 ± 3.02	27.68 ± 3.70	0.562	28.19 ± 3.55	26.77 ± 2.94	0.053
MCHC	32.91 ± 1.35	33.10 ± 1.06	0.489	32.98 ± 1.04	32.97 ± 1.39	0.968	33.03 ± 1.35	32.82 ± 0.92	0.492
MCV	83.88 ± 9.57	85.15 ± 7.44	0.505	85.16 ± 8.26	83.77 ± 9.30	0.452	85.24 ± 9.14	81.47 ± 7.66	**0.048**
PLT	304.04 ± 7.30	274.97 ± 9.31	0.260	283.26 ± 11.11	301.31 ± 12.69	0.472	288.67 ± 114.13	311.80 ± 140.47	0.410
WBC	8.98 ± 5.50	8.05 ± 2.49	0.359	7.43 ± 3.28	9.44 ± 5.31	**0.038**	8.27 ± 4.44	9.89 ± 5.45	0.140
total bilirubin	0.75 ± 1.03	0.70 ± 0.35	0.749	0.55 ± 0.22	0.84 ± 1.07	**0.046**	0.67 ± 0.46	0.94 ± 1.53	0.384
serum creatinine	0.72 ± 0.22	0.82 ± 0.21	**0.031**	0.66 ± 0.19	0.81 ± 0.21	**<0.001**	0.74 ± 0.24	0.79 ± 0.15	0.394
ANC	5.66 ± 4.51	5.45 ± 2.33	0.808	4.54 ± 3.02	6.24 ± 4.27	**0.034**	5.19 ± 3.62	6.79 ± 4.57	0.077
AST	37.97 ± 28.09	39.73 ± 39.89	0.798	37.56 ± 2.82	39.16 ± 3.41	0.811	36.29 ± 30.51	45.32 ± 36.93	0.228
ALT	29.23 ± 26.92	22.07 ± 15.93	0.162	26.78 ± 2.61	26.92 ± 2.26	0.977	24.43 ± 19.01	34.20 ± 34.49	0.188
dNLR	1.73 ± 1.02	2.20 ± 1.08	**0.035**	1.69 ± 1.06	2.01 ± 1.05	0.141	1.77 ± 0.92	2.23 ± 1.37	0.062

Note: Data are presented as mean ± standard deviation. Comparisons between groups were performed using the independent samples *t*-test; Bold values indicate statistical significance (*p* < 0.05); HCT (hematocrit); HGB (hemoglobin); MCV (mean corpuscular volume); MCH (mean corpuscular hemoglobin); MCHC (mean corpuscular hemoglobin concentration); PLT (platelet count); WBC (white blood cell count); ANC (absolute neutrophil count); AST (aspartate aminotransferase); ALT (alanine aminotransferase); dNLR (calculated as ANC/[WBC − ANC]).

**Table 8:** Overall survival according to baseline laboratory parameters (Kaplan–Meier analysis).

Variable	Category (Clinical Cut-Off)	No. of Patients	Median OS (Days)	χ^2^	*p*-Value
HCT	female			1.300	0.254
	Anemia (≤36%)	16	142		
	Normal (>36%)	19	273		
	male				
	Anemia (<39%)	37	211		
	Normal (>39%)	20	152		
HGB	female			0.701	0.402
	Anemia (≤12 g/dL)	18	155		
	Normal (>12 g/dL)	17	273		
	male				
	Anemia (≤13 g/dL)	39	211		
	Normal (>13 g/dL)	18	152		
MCH	Low (<25.6 pg)	23	258	0.640	0.726
	Normal (25.6–32.2 pg)	62	173		
	High (>32.2 pg)	7	199		
MCHC	Low (<32.2 g/dL)	24	173	0.139	0.933
	Normal (32.2–35.5 g/dL)	63	184		
	High (<35.5 g/dL)	5	199		
MCV	Low (<79.4 fL)	25	211	0.515	0.773
	Normal (79.4–94.8 fL)	57	173		
	High (>94.8 Fl)	10	184		
PLT	Low (<150 × 10^9^/L)	7	168	0.217	0.897
	Normal (150–450 × 10^9^/L)	75	209		
	High/Thrombocytosis (>450 × 10^9^/L)	10	168		
WBC	Low (≤3.98 × 10^9^/L)	6	198	0.363	0.834
	Normal (3.98–10.4 × 10^9^/L)	65	184		
	High/Leukocytosis (>10.04 × 10^9^/L)	21	184		
Total bilirubin	Normal (≤1.2 mg/dL)	86	199	1.332	0.248
	High (>1.2 mg/dL)	6	44		
Serum creatinine	Low (≤0.51 mg/dL)	9	168	1.336	0.513
	Normal (0.51–0.95 mg/dL)	69	209		
	High (>0.95 mg/dL)	14	121		
ANC	Low (≤2.0 × 10^9^/L)	11	373	2.434	0.296
	Normal (2.0–7.0 × 10^9^/L)	62	168		
	High / Neutrophilia (>7.0 × 10^9^/L)	19	184		
AST	Normal (≤34.99 U/L)	61	219	3.946	**0.047**
	High (>34.99 U/L)	31	149		
ALT	Normal (≤34.99 U/L)	77	199	0.947	0.330
	High (>34.99 U/L)	15	152		
dNLR	Normal (≤1.63–median)	46	219	1.444	0.229
	High (>1.63–median)	46	168		

Note: Overall survival (OS) was defined as the time from diagnosis to death from any cause or last follow-up. Survival probabilities were estimated using the Kaplan–Meier method and compared between groups using the log-rank test. Clinical cut-off values were defined according to institutional laboratory reference ranges. Bold values indicate statistical significance (*p* < 0.05); HCT (hematocrit); HGB (hemoglobin); MCV (mean corpuscular volume); MCH (mean corpuscular hemoglobin); MCHC (mean corpuscular hemoglobin concentration); PLT (platelet count); WBC (white blood cell count); ANC (absolute neutrophil count); AST (aspartate aminotransferase); ALT (alanine aminotransferase); dNLR (calculated as ANC/[WBC − ANC]).

**Table 9:** Univariable and adjusted multivariable Cox regression models for overall survival.

		Univariable		Multivariable	
Variable	Category (Clinical Cut-Off Reference)	HR (95% CIs)	*p*-Value	HR (95% CIs)	*p*-Value
Age	≤70 vs. >70 (ref)	1.215 (0.766–1.929)	0.408	0.677 (0.390–1.175)	0.165
Sex	Male vs. Female (ref)	1.050 (0.669–1.648)	0.831	-	-
ECOG performance status	≥1 vs. 0 (ref)	0.975 (0.606–1.567)	0.916	1.226 (0.688–2.184)	0.489
Primary tumor sidedness	Right vs. Left (ref)	0.933 (0.561–1.553)	0.791	0.917 (0.533–1.576)	0.753
Liver metastases	Yes vs. No (ref)	1.129 (0.633–2.013)	0.681	1.214 (0.648–2.277)	0.545
Liver comorbidity	Yes vs. No (ref)	1.052 (0.503–2.203)	0.892	-	-
Primary tumor resection	Yes vs. No (ref)	0.609 (0.373–0.995)	**0.048**	1.487 (0.842–2.625)	0.172
First-line treatment	Oxaliplatin-based vs. Fluoropyrimidine-only (ref)	1.189 (0.676–2.081)	0.553	1.112 (0.603–2.053)	0.734
	Irinotecan-based vs. Fluoropyrimidine-only (ref)	1.810 (1.036–3.165)	**0.037**	2.103 (1.162–3.808)	**0.022**
Anemia (WHO sex-specific)	Yes vs. No (ref)	0.812 (0.498–1.323)	0.404	1.420 (0.865–2.306)	0.165
AST	≤34.99 U/L vs. >35.00 U/L	1.588 (1.001–2.519)	**0.050**	1.234 (0.947–1.608)	0.119
dNLR		1.121 (0.904–1.391)	0.297	1.117 (0.849–1.470)	0.429
dNLR tertiles	T2 vs. T1 (ref)	1.983 (1.152–3.413)	**0.014**	-	-
	T3 vs. T1 (ref)	1.378 (0.789–2.408)	0.260	-	-

Note: HR, hazard ratio; CI, confidence interval; Bold values indicate statistical significance (*p* < 0.05); ECOG, Eastern Cooperative Oncology Group performance status; AST, aspartate aminotransferase; HRs and 95% CIs were derived from univariate Cox proportional hazards models, with normal values as the reference category. Overall survival was defined as time from diagnosis to death or last follow-up; dNLR (calculated as ANC/[WBC − ANC]).

## Data Availability

The original contributions presented in this study are included in the article/Supplementary Materials. Further inquiries can be directed to the corresponding authors.

## Supplementary Materials

The following supporting information can be downloaded at: https://www.mdpi.com/article/10.3390/jcm15062319/s1, Table S1: Reference ranges for laboratory parameters (institutional laboratory standards); Table S2: ECOG-PS scale; Table S3: TNM Classification in colon cancer.
