# Supplementary material for: Systemic Inflammation and Survival in Stage IV Colorectal Cancer: A Retrospective Cohort Study
Source: J Clin Med. 2026 Mar 18;15(6):2319. doi: 10.3390/jcm15062319 (PMC13027073; doi:10.3390/jcm15062319)
Supplement: Supplementary file 1 [file jcm-15-02319-s001.zip › jcm-4175657-supplementary.pdf]

Supplementary Materials

Table S1. Reference ranges for laboratory parameters (institutional laboratory standards).

Parameter	Unit	Reference range
HCT (Hematocrit) -female	%	Low (<=36 %)<br>Normal (>36%)
HCT (Hematocrit) - male	%	Low (<39 %)<br>Normal (>39%)
HGB (Hemoglobin) - female	g/dL	Anemia (<=12 g/dL)<br>Normal (>12 g/dL)
HGB (Hemoglobin) - male	g/dL	Anemia (<=13 g/dL)<br>Normal (>13 g/dL)
MCV (Mean Corpuscular Volume)	fL	79.4–94.8
MCH (Mean Corpuscular Hemoglobin)	pg	25.6–32.2
MCHC (Mean Corpuscular Hemoglobin Concentration)	g/dL	32.2–35.5
PLT (Platelets)	$\times 10^9$/L	150–450
WBC (White Blood Cells)	$\times 10^9$/L	3.98–10.04
ANC (Absolute Neutrophil Count)	$\times 10^9$/L	2.0–7.0
Total bilirubin	mg/dL	0.3–1.2
Serum creatinine	mg/dL	0.51–0.95
AST (Aspartate aminotransferase)	U/L	0–34.99
ALT (Alanine aminotransferase)	U/L	0–34.99
dNLR	–	median*

Note: Reference ranges were defined according to institutional laboratory standards. The dNLR was calculated as ANC / (WBC − ANC); no reference range is available for this derived index.

Table S2. ECOG-PS scale.

ECOG-PS score	Description
**0**	Fully active; able to perform all pre-disease activities without functional limitation.
**1**	Ambulatory and capable of light or sedentary tasks; restricted in physically strenuous activity but otherwise independent.
**2**	Ambulatory and independent in self-care; unable to carry out work activities; active and out of bed for more than half of waking hours.
**3**	Limited self-care capacity; confined to bed or chair for more than half of waking hours.
**4**	Completely disabled; fully dependent for self-care; totally confined to bed or chair.
**5**	Death.

ECOG-PS - ECOG Performance Status

Table S3. TNM Classification in colon cancer

Section	Category	Definition
**Primary tumor (T)**	TX	Primary tumor cannot be assessed.
	T0	No evidence of a primary tumor.

Section	Category	Definition
	Tis	Carcinoma in situ (confined to mucosa; no invasion beyond muscularis mucosae).
	T1	Invasion into submucosa.
	T2	Invasion into muscularis propria.
	T3	Extension through muscularis propria into pericolorectal tissues (pericolic/perirectal fat), without reaching the visceral peritoneal surface.
	T4a	Tumor reaches/penetrates the visceral peritoneal surface (serosal involvement where applicable).
	T4b	Direct invasion into, or fixed adherence to, adjacent organs/structures.
**Regional lymph nodes (N)**	NX	Regional lymph nodes cannot be assessed.
	N0	No regional lymph node metastasis.
	N1	Metastasis in 1–3 regional nodes and/or tumor deposits meeting N1c criteria.
	N1a	Metastasis in 1 regional node.
	N1b	Metastasis in 2–3 regional nodes.
	N1c	No positive regional nodes, but tumor deposits in pericolorectal tissues.
	N2	Metastasis in ≥4 regional nodes.
	N2a	Metastasis in 4–6 regional nodes.
	N2b	Metastasis in ≥7 regional nodes.
**Distant metastasis (M)**	M0	No distant metastasis.
	M1	Distant metastasis present (including peritoneal involvement).
	M1a	Metastasis confined to one distant organ/site (no peritoneal metastasis).
	M1b	Metastases in two or more distant organs/sites (no peritoneal metastasis).
	M1c	Peritoneal metastasis (alone or with other distant metastases).
**Anatomic stage grouping**	Stage 0	Tis N0 M0
	Stage I	T1–T2 N0 M0
	Stage IIA	T3 N0 M0
	Stage IIB	T4a N0 M0
	Stage IIC	T4b N0 M0
	Stage IIIA	T1–T2 N1/N1c M0 *or* T1 N2a M0
	Stage IIIB	T3–T4a N1/N1c M0 *or* T2–T3 N2a M0 *or* T1–T2 N2b M0
	Stage IIIC	T4a N2a M0 *or* T3–T4a N2b M0 *or* T4b N1–N2 M0
	Stage IVA	Any T Any N M1a
	Stage IVB	Any T Any N M1b
	Stage IVC	Any T Any N M1c

T = Tumor; N = Nodes; M = Metastasis.
